# Prevalence, Awareness, Treatment and Control of Coexistence of Diabetes and Hypertension in Thai Population

**DOI:** 10.1155/2012/386453

**Published:** 2012-07-19

**Authors:** Siriwat Tiptaradol, Wichai Aekplakorn

**Affiliations:** ^1^Office of Permanent Secretary, Ministry of Public Health, Nonthaburi 11000, Thailand; ^2^Department of Community Medicine, Faculty of Medicine, Ramathibodi Hospital, Mahidol University, Rama VI Road, Rajdevi, Bangkok 10400, Thailand

## Abstract

Diabetes and hypertension are major independent risk factors for cardiovascular and renal diseases; however, prevalence and characteristics of the coexistence in general population is not clear. Data from Thai National Health Examination Survey III were used to estimate the prevalence of coexistence of diabetes and hypertension, and to estimate the proportion of awareness, treatment and control of both conditions. A total of 36,877 (male 17,614 and female 19,263) participants were included in the study. The prevalence of people with diabetes and hypertension was 3.2% (male 2.8% and female 3.6%). Approximately half of the diabetes patients (49.0%, 95%CI 45.6, 52.5) had hypertension, and 14.4% (95%CI 13.0, 16.0) of hypertensive patients had diabetes. After controlling for covariates, factors associated with coexistence of diabetes and hypertension included; age ≥60 years (adjust odds ratio 1.38, 95%CI 1.14, 1.73), having education less than 6 years (1.83, 95%CI 1.03, 3.38) and abdominal obesity (2.49, 95%CI 2.00, 3.10). More than 80% were unaware of having both conditions. Target for control of both glucose and blood pressure among those treated was achieved in only 6.2%. In conclusion, patients with diabetes or hypertension should be promoted to have weight control and screening for the comorbidity.

## 1. Introduction

At present, diabetes and hypertension are among the most common noncommunicable chronic diseases in developed and developing countries around the world [1]. In Thailand, burden of diseases defined as total disability-adjusted life year (DALY) loss attributed to diabetes was 1.7 million years (3.1%) in men and 2.7 million years (6.4%) in women, and to hypertension was 5.5% each in both men and women [2]. Hypertension is commonly found in patients with diabetes and vice versa. People with coexisting diabetes and hypertension are at increased risk of atherosclerosis, retinopathy, renal failure and nontraumatic amputations, and cardiovascular diseases [3, 4]. These conditions not only result in high burden to the patients and family, but also impose a high national health care cost worldwide. In Thailand, the 2004 National Health Examination survey (NHES III) reported the prevalence of 6.8% and 21.0% for diabetes and hypertension, respectively, in Thai population aged ≥15 years [1, 5]. These studies also revealed that more than half of individuals with the each condition were unaware of the condition. Furthermore, the percentages of the patients who were treated and controlled to target levels were substantially low. Although there has been certain information on proportions of patients with both conditions [1], the characteristics of individuals with the coexistence of both conditions have not been clearly identified. Moreover, knowing factors associated with the conditions should be useful for health service and public health action in term management and prevention. The present study aimed to determine the prevalence of coexistence of diabetes and hypertension and the proportions of awareness, treatment, and control of the coexistence of both conditions in Thai population by using data from the NHES III.

## 2. Methods

### 2.1. Study Population

The sampling method and data collection procedures of NHES III were described elsewhere [5, 6]. Briefly, the national health examination survey is a multistage probability sampling of Thai population aged ≥15 years. The sampling unit in each of the four stages of selection included: (1) three provinces in each of the 12 public health regions; (2) nine electoral units (EUs) or villages from urban and rural areas, respectively; and (3) 15 individuals from each EU or village. The final sample size was targeted at 42,120 individuals and the final complete data used for analysis of this study was 36,877 individuals (male 17,614 and female 19,263) aged ≥15 years.

### 2.2. Data Collection

Data on demographic characteristics, lifestyle behavior, history of diabetes and hypertension, and management were interviewed by trained interviewers. Blood pressure was measured by trained field staff according to standard protocol [7]. Three serial measurements of blood pressure, taken 1 minute apart, were obtained using a mercury sphygmomanometer with subjects in the sitting position after 5 min of rest. Weight, height, and waist circumference (WC) were measured by trained field staff using standard procedures and equipment according to the World Health Organization guideline [8]. Subjects wore light clothing but without shoes while their height and weight were measured. WC was measured at a level midway between lower rib margin and iliac crest with a measuring tape on a horizontal plane around the body [8].

Venous blood samples were obtained in the morning after the participants had fasted overnight. Fasting plasma glucose (FPG) was tested using hexokinase enzyme method. Serum cholesterol was measured suing enzymatic methods. The laboratory centers were a standardized central laboratory at the Ministry of Public Health.

### 2.3. Definitions

Blood pressure was the average of two serial blood-pressure measurements with lowest variability in pulse pressure. Hypertension was defined as a systolic (SBP) ≥ 140 mmHg or a diastolic blood pressure (DBP) ≥ 90 mmHg or on medication with blood-lowering agents during the past 2 weeks [7]. Diabetes was defined as FPG ≥ 7.0 mmol/L (≥126 mg/dL) or previous diagnosis and on treatment of diabetes using antiglycemic agents or insulin during the past 2 weeks [1, 9]. Diagnosed diabetes was defined as those who were diagnosed by medical doctors and were on treatment with antiglycemic drugs in the past 2 weeks. Individuals reported on receiving antiglycemic drugs in the past two weeks were considered as on treatment. Individuals on diabetes treatment were considered as being controlled if their FPG was less than 130 mg/dL [10]. Control target of blood pressure was set at SBP < 140 mmHg and DBP < 90 mmHg for hypertensive individuals without diabetes, and SBP < 130 mmHg and DBP < 90 mmHg for those with coexistence of hypertension and diabetes [9]. Asian criteria for obesity was used at cut-off point of BMI ≥ 25 kg/m^2^ and abdominal obesity was defined as WC ≥ 90 cm. for male and ≥80 cm for female [11]. 

### 2.4. Statistical Methods

All the analyses were weighted against the registered 2004 Thai population by public health administration area, urban/rural areas, sex, and 5-year age groups. The analysis was accounted for the complex survey design using “svy” command in Stata software version 10. Subjects were categorized into 4 groups: no diabetes or hypertension, diabetes only, hypertension only, and those with coexistence of diabetes and hypertension. Mean values and 95% confidence interval (CI) for continuous variables were calculated. Prevalence of diabetes, hypertension alone, and the coexistence as well as awareness and treatment of those having the conditions were calculated. The proportion of the controlled was calculated among those who were treated. Multivariable logistic regression was used to examine the association of several independent variables including age, sex, urban/rural and educational level, obesity (BMI ≥ 25 kg/m^2^), and abdominal obesity with the coexistence of diabetes and hypertension.

## 3. Results

The prevalence of diabetes alone, hypertension alone, and the coexistence of both was 3.3% (95%CI 2.8, 4.0), 19.1% (17.6, 20.6), and 3.2% (2.9, 3.6), respectively. The prevalence of the coexistence was higher in women (3.6%, 95%CI 3.2, 4.0) than in men (2.8%, 95%CI 2.4, 3.3) (*P* < 0.05). Approximately half of the patients with diabetes (49.0%, 95%CI 45.6, 52.5) had hypertension, while 14.4% (95%CI 13.0, 16.0) of hypertensive individuals had diabetes.

Subjects with the coexistence were older, having higher BMI, larger waist circumference, higher systolic blood pressure, and higher level of blood total cholesterol compared to those having diabetes alone or hypertension alone. The proportion of participants with high cholesterol (TC ≥ 240 mg/dL) was also highest in those with the coexistence, followed by those with diabetes. People with the coexistence also had highest rates of obesity, followed by those having hypertension alone (Table 1). The prevalence of the coexistence was significantly higher in those with BMI ≥ 25 kg/m^2^ than in those with BMI < 25 kg/m^2^ (6.2% versus 2.0%, resp., *P* < 0.01) as well as higher in those with abdominal obesity than in those without (7.7% versus 1.6%, resp., *P* < 0.01).

**Figure 1:**
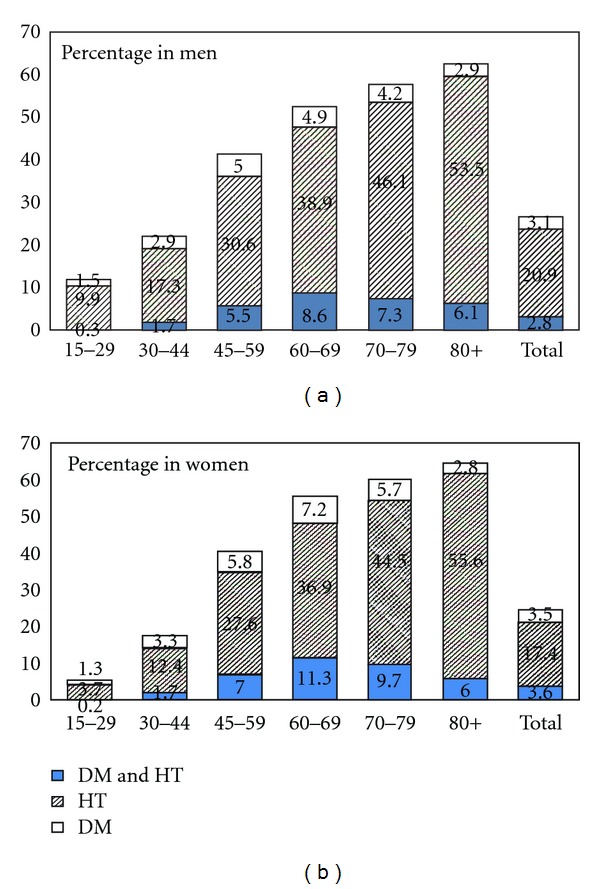
Prevalence of diabetes, hypertension, and coexistence of both conditions in Thai population aged ≥15 years by age group.

Figure 1 shows the prevalence of diabetes alone, hypertension alone and the coexistence of both conditions by age groups. The age-specific prevalence of individuals having either conditions or both conditions increased as age increased and peaked at age 80 and over. Prevalence of coexistence of both conditions was highest in the group 60–69 years (8.6% in men and 11.3% in women).

### 3.1. Urban/Rural and Geographic Difference

The prevalence of the coexistence of both conditions was significantly higher in urban men than rural men (3.9 versus 2.4%, *P* < 0.05) but not significantly different between urban and rural women (3.9 versus 3.5% *P* > 0.05). People in Bangkok had the highest prevalence of the coexistence for both men and women, followed by men in central region and women in the northeastern region (Table 2).

### 3.2. Awareness, Treatment, and Control

Table 3 shows the proportion of awareness diabetes, treatment, and control of fasting plasma glucose, high blood pressure, or both among individuals with coexistence of both conditions. Approximately half of the people with the coexistence were unaware of either one condition and 85% of them were unaware of having both conditions. More people were not treated for hypertension compared to treatment for diabetes. Less than 20% of the individuals were controlled for each condition, and only 6.2% of those with both conditions and treated had their blood glucose and blood pressure under control (Table 3).

### 3.3. Factors Associated with Comorbidity of Diabetes and Hypertension

Table 4 shows factors that were associated with the coexistence compared to those with having either diabetes or hypertension alone. After controlling for potential confounding factors of sociodemographic variables including age, sex, education, urban/rural are as, geographic region, obesity (BMI ≥ 25 kg/m^2^), and abdominal obesity, factors that were associated with the coexistence of both conditions included: age ≥60 years (adjusted odds ratio (OR) 1.38, 95%CI 1.14, 1.70), living in urban area (1.15, 95%CI 1.0, 1.30), having education less than 6 years (1.83, 95%CI 1.03, 3.38), and abdominal obesity (2.49, 95%CI 2.00, 3.10). Note that BMI ≥ 25 kg/m^2^ was not significantly associated with the coexistence in the multivariable model. Additional analysis for multivariable logistic regression was performed using BMI cut-off point at 30 kg/m^2^, instead of at 25 kg/m^2^. The results showed that BMI ≥ 30 kg/m^2^ still was not significantly associated with the coexistence of diabetes and hypertension (adjusted OR 1.17, 95%CI 0.91, 1.50), whereas abdominal obesity remains statistically significance (2.50, 95%CI 2.01, 2.97).

## 4. Discussion

This study added more information on characteristics of individuals with coexistence of diabetes and hypertension in Thai population. A half of the people with diabetes also had hypertension and about 14% of hypertensive patients had diabetes. The prevalence of 3.2% of the coexistence corresponds to an estimated 1.5 million of Thai population having both conditions. After controlling for covariates, patients with the coexistence of diabetes and hypertension were likely to be older, having lower education and abdominal obesity. There were limited data on prevalence of the coexisting conditions in Asian populations. Although the prevalence of diabetes or hypertension in Thai population was comparable to that of other Asian countries [12], it is unclear whether the prevalence of the coexistence of both conditions is different from those of other Asian populations. Further studies of this issue might be warrant.

The coexistence of diabetes and hypertension increases risk of cardiovascular diseases [7, 13], renal complications, and retinopathy [7]. Randomized control trial studies demonstrated that aggressive control of blood pressure and diabetes could improve the CVD outcomes [14]. Therefore, early detection and control of the coexisting of the disease are essential. However, the present study revealed that awareness of the coexistence of both conditions was very low with less than 30% of individuals with both conditions being treated and less than 10% of the conditions being simultaneously controlled. The findings suggested that over 1 million individuals with both conditions were not detected and treated for their conditions.

Awareness of diabetes, hypertension and coexistence of both were lower in men than in women. This might reflect that men are less likely to seek health care and less health concern compared to women. This finding is consistent with the national survey data showing the higher health care utilization among women than men [15]. This finding suggests that health education program should be focused more on men.

The high proportion of having hypertension in patients with diabetes (78.4%) had been reported in a study using the data from the Thai diabetes registry [16]. The proportion found in this study was lower than that of the registry data, because the cases in the registry were from the tertiary care hospitals, where severe cases of diabetes were more likely to be found. However, the proportion of hypertension in patients with diabetes in this study was higher than that of the United Kingdom prospective diabetes study (UKPDS), which reported the proportion of 39% [17, 18]. The proportion of diabetes among hypertensive patients in this study was slightly lower than what found in the US where the proportion of individuals with hypertension having diabetes was 19.8% [19]. These findings are consistent with previous review [13] in that approximately half of the diabetes patients have hypertension, and hypertension was about twice as frequent in individuals with diabetes compared to those without diabetes.

BMI is a measure of total obesity and abdominal obesity is an indicator for abdominal fat deposition. This study found that abdominal obesity was a stronger risk factor for the coexistence of diabetes and hypertension. This finding was consistent with many studies and review on evidence of WC being a stronger risk factor for cardiovascular diseases and metabolic syndrome as compared to BMI [20–23]. However, some recent studies argued that the evidence to replace BMI with waist circumference for clinical or public health practice has not been strong enough [24, 25]. More research, particularly in Asian populations, is needed to confirm whether measuring abdominal obesity should replace BMI in surveillance of cardiovascular risk factors.

Other factors associated with the coexistence included education level less than 6 years. In a previous study we also reported that hypertension was more prevalent among those with lower education [5]. It is possible that those with lower education were at high risk, because they might have worse unhealthy lifestyles. In those with inadequate control either diabetes or hypertension might lead to the development of the other condition. A study reported that patients with hypertension are 2.5 times more likely to develop diabetes compared to those with normotension [13]. 

The strength of this study includes having large sample size and being a representative data that allows the analysis of each condition and the coexistence. Some limitations of the present study should be mentioned. First, the present study might underestimate the true prevalence of diabetes, since only FPG was used. Two-hour glucose tolerance test was reported to be more accurate in detection of undiagnosed diabetes compared to measurement of fasting plasma glucose; however, FPG test was more feasible to perform in field survey [26]. Second, the measurement of blood pressure could be subject to overestimation due to the effect of white coat hypertension. However, the measurement was done according to the standard protocol for blood-pressure measurement and the procedure was done in the community, so this effect might be minimal. Third, the cross-sectional design might preclude drawing conclusion about cause effect relationship between risk factors and the comorbidity.

The implication of this study is that the health care system should be designed to strengthen comprehensive health care of coexistence of diabetes and hypertension in order to increase detection, treatment, and control of those affected. In addition, individuals with obesity or those with low educational levels were at higher risk and should be targeted.

## 5. Conclusion

Patients with diabetes or hypertension should be screened for coexistence of another condition, and they should be promoted to have weight control in order to prevent the comorbidity. More stringent control of plasma glucose and blood pressure among these patients should be applied.

## Figures and Tables

**Table 1:** Characteristics of participants with diabetes, hypertension and coexistence of both conditions in Thai population aged ≥15 years, 2004.

	No DM and no HT (*n* = 20,906)	DM only (*n* = 1,715)	HT only (*n* = 11,708)	DM and HT (*n* = 2,548)	*P* value
Male	*n* = 9,867	*n* = 724	*n* = 5,923	*n* = 1,100	

Age (yr)	35.7 (35.3, 36.2)	45.3 (42.5, 48.1)	48.3 (47.1, 49.4)	54.9 (52.9, 57.0)	<0.01
BMI (kg/m^2^)	22.1 (21.9, 22.3)	23.3 (22.4, 24.2)	24.2 (23.8, 24.7)	27.0 (25.5, 28.5)	<0.01
Waist (cm)	77.2 (76.6, 77.7)	81.8 (80.3, 83.4)	82.9 (81.8, 84.1)	90.9 (87.0, 94.8)	<0.01
FPG (mg/dL)	89.2 (88.2, 90.2)	170.9 (165.9, 175.9)	91.4 (90.1, 92.7)	185.1 (173.5, 196.6)	<0.01
SBP (mmHg)	114.0 (113.5, 114.6)	115.6 (113.9, 117.4)	137.8 (136.3, 139.4)	139.0 (137.3, 140.8)	<0.01
TC (mg/dL)	185.8 (183.5, 188.2)	194.4 (178.9, 209.8)	198.0 (194.1, 201.8)	201.9 (181.1, 222.8)	<0.01
TC ≥ 240 (%)	11.6 (10.4, 12.9)	20.1 (15.5, 25.5)	18.5 (15.5, 22.0)	38.0 (31.3, 45.0)	<0.01
BMI ≥ 25 (%)	17.9 (16.2, 19.7)	30.5 (23.6, 38.4)	36.6 (32.7, 40.6)	57.2 (47.2, 66.7)	<0.01
Abdominal obesity (%)	11.0 (9.8, 12.3)	24.9 (19.2, 31.6)	26.6 (23.3, 30.2)	50.4 (39.8, 61.0)	<0.01

Female	*n* = 11,039	*n* = 991	*n* = 5,785	*n* = 1,448	<0.01

Age (yr)	36.1 (35.6, 36.6)	49.1 (47.1, 51.2)	53.9 (53.0, 54.8)	56.6 (55.4, 57.9)	<0.01
BMI (kg/m^2^)	23.3 (23.1, 23.4)	25.2 (24.3, 26.2)	25.6 (25.1, 26.3)	29.4 (28.3, 30.5)	<0.01
Waist (cm)	75.2 (74.6, 75.8)	81.7 (79.2, 84.2)	80.7 (79.4, 82.0)	90.7 (88.7, 92.6)	<0.01
FPG (mg/dL)	86.6 (85.6, 87.5)	181.7 (168.5, 194.9)	88.8 (87.6, 89.9)	172.8 (163.4, 182.2)	<0.01
SBP (mm Hg)	110.9 (110.3, 111.5)	113.5 (112.1, 115.0)	136.6 (135.0, 138.1)	144.9 (143.3, 146.5)	<0.01
TC (mg/dL)	194.6 (192.2, 197.0)	195.1 (176.6, 213.5)	203.9 (198.8, 209.0)	221.1 (213.0, 229.2)	<0.01
TC ≥ 240 (%)	14.2 (12.9, 15.6)	27.8 (19.0, 38.6)	21.0 (18.2, 24.1)	42.2 (38.96, 45.5)	<0.01
BMI ≥ 25 (%)	30.1 (28.6, 31.7)	45.08 (37.4, 53.0)	52.2 (47.0, 57.2)	63.6 (56.7, 69.9)	<0.01
Abdominal obesity (%)	31.0 (29.0, 33.0)	51.1 (43.3, 58.9)	51.4 (46.6, 56.2)	84.3 (81.0, 84.3)	<0.01

Data were mean and percent (%) as specified; DM: diabetes; HT: hypertension; BMI: body mass index; FPG: fasting plasma glucose; SBP: systolic blood pressure; TC: total cholesterol; Abdominal obesity, waist circumference ≥90 cm in men and ≥80 cm in women.

**Table 2:** Prevalence (95%CI) of diabetes, hypertension and coexistence of both conditions by area of residence and region in Thai population aged ≥15 years.

	DM only % (95%CI)	HT only % (95%CI)	Coexistence of both % (95%CI)
Male			
Urban	3.8 (3.1, 4.7)	22.8 (20.6, 25.2)	3.9 (3.4, 4.5)
Rural	2.8 (2.1, 3.9)	20.2 (18.1, 22.6)	2.4 (1.9, 3.0)
Region			
Central	2.8 (2.3, 3.6)	22.2 (19. 5, 25.3)	3.2 (2.7, 3.8)
Northeastern	3.7 (2.3, 5.1)	18.6 (14.9, 23.0)	2.6 (1.7, 3.8)
North	2.4 (1.8, 3.3)	25.1 (21.2, 29.4)	2.6 (2.0, 3.4)
South	2.4 (1.4, 4.3)	17.9 (15.6, 20.5)	2.2 (1.6, 3.0)
Bangkok	4.3 (3.1, 5.8)	17.5 (14.3, 21.1)	5.2 (3.8, 7.0)
Female			
Urban	3.4 (2.9, 4.0)	18.0 (16.5, 19.6)	3.9 (3.5, 4.3)
Rural	3.6 (3.0, 4.5)	17.2 (15.8, 18.7)	3.5 (3.1, 4.0)
Region			
Central	3.2 (2.5, 4.1)	19.2 (17.3, 21.3)	3.4 (3.0, 4.0)
North eastern	4.5 (3.3, 6.1)	16.0 (13.8, 18.4)	3.9 (3.1, 4.8)
North	3.0 (2.4, 3.7)	19.2 (16.1, 22.6)	3.6 (2.9, 4.4)
South	2.9 (2.3, 3.7)	15.0 (13.3, 16.8)	3.0 (2.2, 4.1)
Bangkok	3.9 (2.6, 5.9)	14.6 (12.9, 16.5)	4.4 (3.6, 5.4)

**Table 3:** Proportion of awareness, treated, and controlled of those having diabetes, hypertension, and coexistence of both conditions in Thai population aged ≥15 years.

	Male % (95%CI)	Female % (95%CI)	Total % (95%CI)
Diabetes only			
Aware	43.3 (37.1, 49.8)	61.9 (55.4, 68.0)	54.1 (48.3, 59.8)
Treated	40.8 (34.9, 47.0)	60.2 (53.9, 66.2)	52.1 (46.5, 57.6)
Controlled among treated	33.2 (27.1, 40.0)	38.1 (32.4, 44.0)	36.5 (32.4, 40.7)
Hypertension only			
Aware	43.0 (36.7, 49.5)	52.3 (46.1, 58.5)	48.4 (43.0, 53.9)
Treated	36.4 (30.5, 42.7)	46.0 (39.7, 52.1)	42.0 (36.9, 47.3)
Controlled among treated	12.2 (8.3, 17.5)	16.5 (11.7, 22.7)	14.9 (11.4, 19.3)
Coexistence of both			
Aware	26.6 (21.9, 32.0)	39.3 (33.5, 45.3)	34.0 (29.4, 38.9)
Treated	22.4 (18.6, 26.7)	34.6 (29.0, 40.7)	29.5 (25.3, 34.1)
Controlled among treated	2.6 (1.4, 4.6)	7.8 (4.5, 13.4)	6.2 (3.8, 9.9)

Controlled target of FPG for those with diabetes alone: FPG at <130 mg/dL.

Controlled target of blood pressure for those with hypertension alone: SBP/DBP at <140/90 mm Hg.

Controlled target of blood pressure for those having coexistence of diabetes and hypertension: SBP/DBP at <130/80 mm Hg.

**Table 4:** Factors associated with coexistence of hypertension and diabetes compared to those having either diabetes or hypertension alone.

	Unadjusted OR	Adjusted OR^∗^
Female (versus male = 0)	1.48 (1.26, 1.73)	1.02 (0.85, 1.20)
Age ≥60 years (aged 15–<59 yrs = 0)	1.50 (1.30, 1.74)	1.38 (1.14, 1.70)
Urban (versus rural = 0)	1.22 (1.03, 1.45)	1.15 (1.00, 1.30)
Education (versus no formal education = 0)		
less than 6 year	1.93 (1.09, 3.42)	1.83 (1.03, 3.38)
secondary	0.92 (0.52, 1.63)	0.96 (0.54, 1.72)
university	1.06 (0.54, 2.06)	1.06 (0.56, 2.01)
BMI ≥ 25 kg/m^2^ (versus BMI < 25 kg/m^2^ = 0)	1.80 (1.54, 2.10)	1.07 (0.86, 1.34)
Abdominal obesity (versus no = 0)	2.58 (2.18, 3.06)	2.49 (2.00, 3.10)

^∗^Adjusted OR controlled for age, area of residence, education, geographic region, BMI status, and abdominal obesity; abdominal obesity: waist circumference ≥90 cm in male and ≥80 cm in female.
